# Meta-atoms: From Metamaterials to Metachips

**DOI:** 10.34133/research.0587

**Published:** 2025-01-10

**Authors:** Hao Chi Zhang, Sen Gong, Le Peng Zhang, Yaxin Zhang, Tie Jun Cui

**Affiliations:** ^1^ State Key Laboratory of Millimeter Waves, Southeast University, Nanjing 211189, China.; ^2^ University of Electronic Science and Technology of China, Chengdu 610054, China.

## Abstract

Electromagnetic (EM) metamaterials represent a cutting-edge field that achieves anomalously macroscopic properties through artificial design and arrangement of microstructure arrays to freely manipulate EM fields and waves in desired ways. The unit cell of a microstructure array is also called a meta-atom, which can construct effective medium parameters that do not exist in traditional materials or are difficult to realize with traditional technologies. By deep integration with digital information, the meta-atom is evolved to a digital meta-atom, leading to the emergence of information metamaterials. Information metamaterials break the inherent barriers between the EM and digital domains, providing a physical platform for controlling EM waves and modulating digital information simultaneously. The concepts of meta-atoms and metamaterials are also introduced to high-frequency integrated circuit designs to address issues that cannot be solved by traditional methods, since lumped-parameter models become unsustainable at microscopic scales. By incorporating several meta-atoms to form a metachip, precise manipulation of the EM field distribution can be achieved at microscopic scales. In this perspective, we summarize the physical connotations and main classifications of meta-atoms and briefly discuss their future development trends. Through this article, we hope to draw more research attention to explore the potential values of meta-atoms, thereby opening up a broader stage for the in-depth development of metamaterials.

Metamaterials broadly refer to the creation of a material-like system with anomalously macroscopic electromagnetic (EM) properties through artificially designed microstructures. They represent a new material construction paradigm that has emerged in the scientific community in recent years and has garnered widespread attention in engineering applications. By designing the unit cells of a microstructure array, which are also called meta-atoms, and their distributions, a metamaterial enables abnormal EM constitutive parameters, such as negative permittivity, negative permeability, and zero refractive index, that are difficult to realize by traditional technologies or do not even exist in nature. Metamaterials can flexibly manipulate the characteristics of EM fields and waves through meta-atoms and array structures, enabling novel physical phenomena such as negative refraction, zero refraction, perfect cloaking, and superresolution lenses [1–3]. Subsequently, by integrating with switching components such as diodes, digital meta-atoms are proposed, which possess the ability to directly represent and manipulate EM waves using digital coding sequences, pushing to establish a new area of information metamaterials [4–6]. Information metamaterials have become an important breakthrough in fusing EM physics and digital information, transitioning metamaterials from structural materials to dynamic information processing systems. They have pioneered the appearance of reconfigurable intelligent surfaces for 6th-generation/beyond-6th-generation mobile communication, which is recognized as one of the potential key technologies for 6th-generation communications [7]. This provides a novel approach for the intersection of metamaterials with various other fields.

In recent years, metamaterials have gradually evolved from low frequencies to high frequencies and from macroscales to microscales (centimeter to nanometer scales). Notably, their development in the fields of millimeter-wave and terahertz-frequency chips has been particularly remarkable. Relevant research has found that introducing artificial microstructural units or arrays (meta-atoms) into millimeter-wave/terahertz chip circuits can finely regulate the EM field distribution in the chip circuits at the microscopic scale. This approach is expected to address some bottlenecks in physics arising from size and wavelength-scale effects in high-frequency chips [8–10], enabling effective suppression of parasitic modes, reduction of adjacent crosstalk, and enhancement of interactions [11–20]. Therefore, a system combining these meta-atoms with chip circuits is referred to as a metachip (Figure). Metachips also represent an expansion in the field of metamaterials, shifting the operating mode of metamaterials and metasurfaces from inter-unit coupling and collective interaction within arrays to intra-unit and unit–circuit interaction on the chip. The issues involved in metachips are different from those in research on 3-dimensional and 2-dimensional arrays of macroscopic metamaterials and metasurfaces. Compared with traditional metamaterials, metachips belong to research in the microscopic domain of individual microstructures, focusing on the local behavior (microscopic distribution) of meta-atoms in regulating the local EM field distribution at the microscopic level. Stronger field localization can reduce the EM interaction area; thus, the fields can be controlled by just several meta-atoms. Typically, the number of meta-atoms used in metachips is often less than 10.

**Figure.**
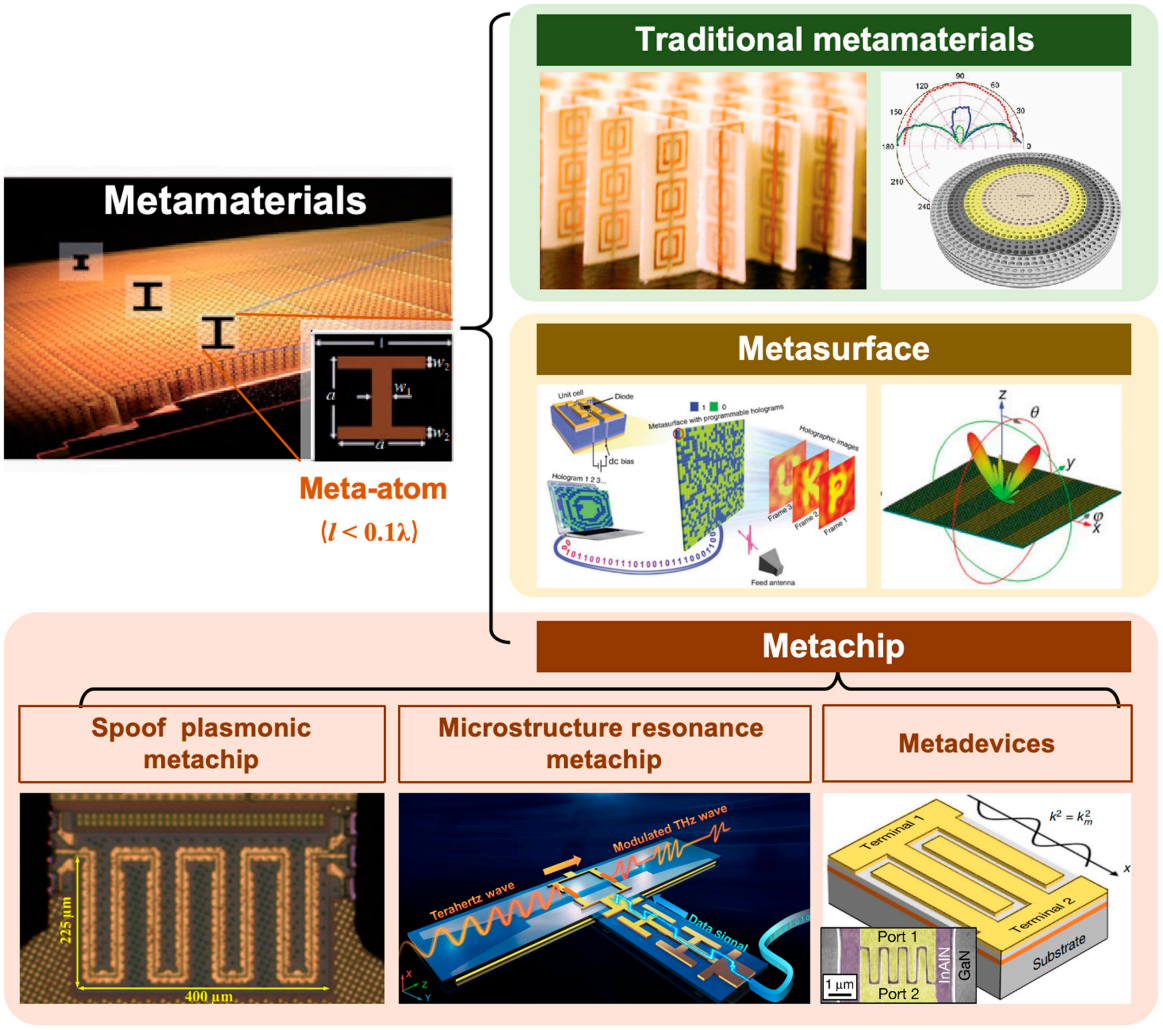
Classification diagram of metamaterials and metachips [1–6,11,15,20].

Based on the objects of integration, metachips can be broadly classified into 3 categories. The first category includes dispersive transmission-type metachips, exemplified by spoof plasmonic metachips [11–13]. This type of metachip is achieved by integrating 1-dimensional periodically arranged meta-atoms with signal transmission pathways. By employing the quasi-uniform collective EM response of the meta-atom, transmission modes with high confinement factors and low dispersion are established. These transmission modes, such as spoof surface plasmon polaritons, can replace the traditional quasi-transverse electromagnetic mode transmission. This replacement effectively suppresses the level of adjacent crosstalk in on-chip channels and dramatically increases the bandwidth density for parallel transmissions. The second category includes resonant-type metachips based on field distribution mode construction, exemplified by microstructure resonance metachips [14–17]. This type of metachip is realized by integrating transistors with meta-atoms through joint design. By leveraging the locally enhanced on-chip field distribution created by the microstructure resonance mode, it enhances the interaction with the transistors and suppresses parasitic parameters generated by higher-order modes. This approach increases the modulation depth and enables high-speed modulations. The third category includes metadevices based on carrier distribution construction [18–20]. For example, metadevices can combine customized meta-atoms with transistor electrodes. By using meta-atoms to finely manipulate the fields in the active region at deep subwavelength scales, they reduce the parasitic capacitance, thereby increasing the operating frequency of switching-type metadevices. Additionally, they adjust the carrier distribution to enhance the power handling capability of the devices.

From above discussions, we note that metachips have abandoned the traditional integrated circuit design approach that solely relies on circuits based on lumped parameters. By introducing microscopic meta-atoms, we achieved precise EM field manipulation capabilities in chip scenarios, thereby possessing potential performance advantages such as higher integration and lower power consumption. Similar to the early development of metamaterials, the current metachips are primarily focused on the regulation of EM properties in the analog domain. Based on the development trajectory of metamaterials, it can be foreseen that the deep digitization of meta-atoms will further extend to microscopic metachips, giving rise to digital metachips. In the future, digital metachips will directly build a bridge between the digital coding domain and the EM physical domain, extending the key technologies of digital meta-atoms to chip scenarios. This is not merely a simple scaling of physical size but rather a marked advancement that endows chip circuits at the microscopic scale with the ability to digitally regulate EM field distributions, making them a vital candidate for achieving deep integration between the digital and EM domains in future microscopic scenarios.

Through decades of efforts by peers, metamaterials have undergone multiple expansions in scope and concept and have penetrated into the microscopic realm (meta-atoms). In the future, while further deepening theoretical innovations, meta-atoms will place a greater emphasis on breakthroughs at the system level that combine macroscopic and microscopic perspectives. This benefits not only from the increasingly sophisticated research on metamaterials at the macroscopic level over the years but also from the gradual breakthroughs made in metachips at the microscopic level. Therefore, it will become an important trend to break the inherent barriers between macroscopic metamaterials and microscopic metachips and realize a fully integrated meta-system with deep fusion of macrosystems and micro-meta-atoms at a higher level. This meta-system will provide a platform solution with more powerful EM control capabilities; deconstruct the traditional hierarchical architecture of information systems, namely, components–subassemblies–systems; and replace it with a complete functional system directly constructed from meta-atoms of various scales. This delayered and fully meta-architecture leverages the powerful EM control capabilities of meta-atoms across macroscopic and microscopic scales. It is expected to directly customize the design of various types of meta-atoms based on the functional requirements of the target system, thereby enabling on-demand design of any EM functionality. This meta-system architecture has the ability of deep digital–analog integration and can directly manipulate digital information in the EM domain, effectively solving the inherent problems of traditional information system architectures, such as the small bandwidth of analog-to-digital conversion, poor confidentiality, low efficiency of digital signal processing, high system costs, and high complexity.

In summary, the new meta-system architecture is expected to directly use EM functional requirements as the customized design criterion, meta-atoms across all scales as the core technology, and higher degrees of freedom in EM manipulation as the main advantage. Ultimately, it aims to construct EM systems with complete information functionality in the form of fully integrated meta-atoms, in order to adapt to continuous pursuit of complexity, intelligence, and integration in information technology. This innovative underlying design approach is likely to trigger disruptive effects and become one of the important driving forces behind a new round of technological revolution. Furthermore, it has the potential to become a sustained driving force for transforming EM information technology and influencing the rules governing the construction of the EM world.

## References

[1] Shelby RA, Smith DR, Schultz S. Experimental verification of a negative index of refraction. Science. 2001;292(5514):77–79. 11292865 10.1126/science.1058847

[2] Liu R, Ji C, Mock JJ, Chin JY, Cui TJ, Smith DR. Broadband ground-plane cloak. Science. 2009;323(5912):366–369. 19150842 10.1126/science.1166949

[3] Jiang WX, Qiu CW, Han TC, Cheng Q, Ma HF, Zhang S, Cui TJ. Broadband all-dielectric magnifying lens for far-field high-resolution imaging. Adv Mater. 2013;25(48):6963–6968. 24352983 10.1002/adma.201303657

[4] Cui TJ, Liu S, Zhang L. Information metamaterials and metasurfaces. J Mater Chem C. 2017;5(15):3644–3668.

[5] Cui TJ, Qi MQ, Wan X, Zhao J, Cheng Q. Coding metamaterials, digital metamaterials and programmable metamaterials. Light Sci Appl. 2014;3: Article e218.

[6] Li L, Cui TJ, Ji W, Liu S, Ding J, Wan X, Li YB, Jiang M, Qiu C-W, Zhang S. Electromagnetic reprogrammable coding-metasurface holograms. Nat Commun. 2017;8: Article 197. 28775295 10.1038/s41467-017-00164-9 PMC5543116

[7] Tang W, Chen MZ, Dai JY, Zeng Y, Zhao X, Jin S, Cheng Q, Cui TJ. Wireless communications with programmable metasurface: New paradigms, opportunities, and challenges on transceiver design. IEEE Wirel Commun. 2020;27(2):180–187.

[8] Chau R, Doyle B, Datta S, Kavalieros J, Zhang K. Integrated nanoelectronics for the future. Nat Mater. 2007;6(11):810–812. 17972935 10.1038/nmat2014

[9] Shen PC, Su C, Lin YX, Chou AS, Cheng CC, Park JH, Chiu MH, Lu AY, Tang HL, Tavakoli MM, et al. Ultralow contact resistance between semimetal and monolayer semiconductors. Nature. 2021;593(7858):211–217. 33981050 10.1038/s41586-021-03472-9

[10] Sengupta K, Nagatsuma T, Mittleman DM. Terahertz integrated electronic and hybrid electronic–photonic systems. Nat Electron. 2018;1(12):622–635.

[11] Liang Y, Yu H, Zhang HC, Yang C, Cui TJ. On-chip sub-terahertz surface plasmon polariton transmission lines in CMOS. Sci Rep. 2015;5: Article 14853. 26445889 10.1038/srep14853 PMC4597218

[12] Liang Y, Yu H, Wang H, Zhang HC, Cui TJ. Terahertz metadevices for silicon plasmonics. Chip. 2022;1(4): Article 100030.

[13] Garcia-Vidal FJ, Fernandez-Dominguez AI, Martin-Moreno L, Zhang HC, Tang W, Peng R, Cui TJ. Spoof surface plasmon photonics. Rev Mod Phys. 2022;94(2): Article 025004.

[14] Zeng H, Liang H, Zhang Y, Wang L, Liang S, Gong S, Li Z, Yang Z, Zhang X, Lan F, et al. High-precision digital terahertz phase manipulation within a multichannel field perturbation coding chip. Nat Photonics. 2021;15(10):751–757.

[15] Zhang Y, Ding K, Zeng H, Kou W, Zhou T, Zhou H, Gong S, Zhang T, Wang L, Liang S, et al. Ultrafast modulation of terahertz waves using on-chip dual-layer near-field coupling. Optica. 2022;9(11):1268–1257.

[16] Gong S, Zeng H, Zhang Q, Bi C, Wang L, Zhou T, Yang Z, Zhang Y, Meng F, Zhang Z, et al. Terahertz meta-chip switch based on C-ring coupling. Nanophotonics. 2022;11(9):2037–2044. 39633924 10.1515/nanoph-2021-0646 PMC11501811

[17] Gong S, Ping D, Bi C, Zhang Z, Liang S, Wang L, Zeng H, Ding K, Dong Y, Zhou H, et al. High-performance direct terahertz modulator based on resonance mode transformation for high-speed wireless communication. Appl Phys Lett. 2022;121(23): Article 231104.

[18] Ritzkowsky F, Yeung M, Bebeti E, Gebert T, Matsuyama T, Budden M, Mainz RE, Cankaya H, Berggren KK, Rossi GM, et al. On-chip petahertz electronics for single-shot phase detection. Nat Commun. 2024;15: Article 10179. 39580474 10.1038/s41467-024-53788-z PMC11585632

[19] Mohammad SN, Armin J, Perera N, Zhu M, Santoruvo G, Matioli E. Nanoplasma-enabled picosecond switches for ultrafast electronics. Nature. 2020;579:534–539. 32214267 10.1038/s41586-020-2118-y

[20] Nikoo MS, Matioli E. Electronic metadevices for terahertz applications. Nature. 2023;614(7948):451–455. 36792737 10.1038/s41586-022-05595-z

